# Dietary fatty acids and mortality risk from heart disease in US adults: an analysis based on NHANES

**DOI:** 10.1038/s41598-023-28738-2

**Published:** 2023-01-28

**Authors:** Yutang Wang, Yan Fang, Paul K. Witting, Fadi J. Charchar, Christopher G. Sobey, Grant R. Drummond, Jonathan Golledge

**Affiliations:** 1 Discipline of Life Science, Institute of Innovation, Science and Sustainability, Federation University Australia, University Drive, Mt Helen, Ballarat, VIC 3350 Australia; 2 Molecular Biomedicine Theme, Faculty of Medicine and Health, Charles Perkins Centre, School of Medical Sciences, The University of Sydney, Sydney, NSW Australia; 3 Department of Microbiology, Anatomy, Physiology and Pharmacology, Centre for Cardiovascular Biology and Disease Research, School of Agriculture, Biomedicine and Environment, La Trobe University, Melbourne, VIC Australia; 4 Queensland Research Centre for Peripheral Vascular Disease, College of Medicine and Dentistry, James Cook University, Townsville, QLD Australia; 5 Department of Vascular and Endovascular Surgery, The Townsville University Hospital, Townsville, QLD Australia

**Keywords:** Cardiology, Medical research, Risk factors

## Abstract

We investigated the association of dietary intake of major types of fatty acids with heart disease mortality in a general adult cohort with or without a prior diagnosis of myocardial infarction (MI). This cohort study included US adults who attended the National Health and Nutrition Examination Surveys from 1988 to 2014. Heart disease mortality was ascertained by linkage to the National Death Index records through 31 December 2015. Cox proportional hazards models were used to estimate hazard ratios (HRs) and 95% confidence intervals (CIs) of fatty acid intake for heart disease mortality. This cohort included 45,820 adults among which 1,541 had a prior diagnosis of MI. Participants were followed up for 532,722 person-years (mean follow-up, 11.6 years), with 2,313 deaths recorded from heart disease being recorded. Intake of saturated (SFAs) and monounsaturated fatty acids (MUFAs) was associated with heart disease mortality after adjustment for all the tested confounders. In contrast, a 5% higher calorie intake from polyunsaturated fatty acids (PUFAs) was associated with a 9% (HR, 0.91; 95% CI 0.83–1.00; *P* = 0.048) lower multivariate-adjusted risk of heart disease mortality. Sub-analyses showed that this inverse association was present in those without a prior diagnosis of MI (HR,0.89; 95% CI 0.80–0.99) but not in those with the condition (HR, 0.94; 95% CI 0.75–1.16). The lack of association in the MI group could be due to a small sample size or severity and procedural complications (*e.g.*, stenting and medication adherence) of the disease. Higher PUFA intake was associated with a favourable lipid profile. However, further adjustment for plasma lipids did not materially change the inverse association between PUFAs and heart disease mortality. Higher intake of PUFAs, but not SFAs and MUFAs, was associated with a lower adjusted risk of heart disease mortality in a large population of US adults supporting the need to increase dietary PUFA intake in the general public.

## Introduction

Heart disease is the leading cause of death in the US and responsible for about one third of all deaths; about 697,000 people died from heart disease in 2020 alone^1^. Heart disease was estimated to cost the US about $229 billion each year from 2017 to 2018^2^. Therefore, research identifying robust heart disease risk factors is critical for prevention^3^. The American Heart Association has recommended lowering dietary saturated fatty acid (SFA) intake as important for reducing cardiovascular events since 1961^4,5^. Advice to substitute SFAs with monounsaturated (MUFAs) and polyunsaturated fatty acids (PUFAs) has been a cornerstone of worldwide dietary guidelines over the past 60 years^4,5^ as unsaturated fatty acids protect against cardiovascular disease in many studies^6–11^.

To date, 11 published randomised controlled trials (RCTs)^8–21^ have investigated the effect of dietary PUFAs on cardiovascular disease events and mortality (Supplementary Table 1). The American Heart Association recommendations are supported by a number of RCTs that showed that replacing SFAs with PUFAs reduced the risk of myocardial infarction (MI) and coronary heart disease death^8–11^. It has, however, been unclear what is the relative importance of reducing SFA intake versus increasing PUFA intake in reducing cardiovascular risk. Notably, a number of meta-analyses suggest that reducing SFA intake does not protect against heart disease mortality^22,23^. Of note, these RCTs used very high doses of PUFAs in the intervention group (mean percentage of calories from PUFAs, 17.2% versus 5.7% in the control group; Supplementary Table 2), which do not likely represent dietary intake in the general public.

The protective effect of PUFAs has also been challenged in recent years. For example, the reanalysis of the Sydney Diet Heart Study in 2013^20^ and the Minnesota Coronary Survey in 2016^21^ showed that increasing intake of PUFAs may increase the risk of coronary heart disease mortality. The importance of MUFA intake in heart disease is also controversial. MUFAs have been regarded as protective against heart disease^3,5^; however, a meta-analysis of six prospective cohort studies^24^ found that MUFA intake had no influence on coronary heart disease events. Moreover, whether increasing PUFAs could be a secondary preventive approach against heart disease is controversial. The Medical Research Council Soy Oil Trial^13^ and the Diet and Reinfarction Trial^17^ showed that increased PUFA intake did not protect patients with a past history of MI; whereas the Oslo Diet-Heart Study^8^ showed that increased PUFA intake reduced the risk of recurrent MI, angina or sudden death in those with pre-existing coronary heart disease. In addition, the Rose Corn Oil Trial^12^ and the Sydney Diet Heart Study^14,20^ reported that higher intake of PUFAs increased the risk of death in patients with pre-existing coronary heart disease.

Given this inconsistent evidence, a large cohort study with an extensive follow-up period is required to resolve the importance of intake of fatty acids in the general population. The current study aimed to investigate the association between fatty acid intake and heart disease mortality in a general cohort of 45,820 US adults who participated in the National Health and Nutrition Examination Survey (NHANES) from 1988 to 2014, stratified by a prior diagnosis of MI.

## Results

### General characteristics

This study included 45,820 US adults with a mean (SD) age of 45.7 (17.3) years. A total of 1,541 participants had a prior diagnosis of MI. The mean percentage of calories from usual intake of SFAs, MUFAs, and PUFAs in this cohort was 11.0%, 12.4%, and 7.4%, respectively, and the intake was similar between those with and without MI (11.0% versus 11.0%, 12.5% versus 12.4%, and 7.5% versus 7.4%, respectively; Table 1). Compared with those without a prior diagnosis of MI, people with the condition were older, more likely to be males, obese and smokers, had lower income and education, and a higher prevalence of hypertension, hypercholesterolemia, and diabetes (Table 1).

**Table 1:** Baseline characteristics of 45,820 participants.

Variables	Prior diagnosis of MI		Overall	*P* value^a^
	Yes	No		
Sample size	1,541	44,279	45,820	NA
Age, y, mean (SD)	65.3 (13.1)	45.0 (17.0)	45.7 (17.3)	< 0.001
SFA,^b^ g/d, median (IQR)	21.3 (15.7–28.2)	24.0 (17.9–31.8)	24.0 (17.8–31.7)	< 0.001
MUFA,^b^ g/d, median (IQR)	23.9 (18.4–31.7)	27.3 (20.5–35.8)	27.2 (20.4–35.7)	< 0.001
PUFA,^b^ g/d, median (IQR)	14.5 (10.5–19.2)	16.1 (12.0–21.2)	16.1 (11.9–21.2)	< 0.001
Protein,^b^ g/d, median (IQR)	71 (56–88)	78 (62–97)	78 (62–96)	< 0.001
Carbohydrate,^b^ g/d, median (IQR)	224 (173–274)	247 (196–309)	246 (195–308)	< 0.001
Caloric % from SFA,^b^ %, mean (SD)	11.0 (2.6)	11.0 (2.6)	11.0 (2.6)	0.998
Caloric % from MUFA,^b^ %, mean (SD)	12.5 (2.7)	12.4 (2.7)	12.4 (2.7)	0.092
Caloric % from PUFA,^b^ %, mean (SD)	7.5 (2.1)	7.4 (2.1)	7.4 (2.1)	0.039
Sex (male), n (%)	1,017 (66.0)	20,421 (46.1)	21,438 (46.8)	< 0.001
Ethnicity, n (%)				
Non-Hispanic white	941 (61.1)	19,216 (43.4)	20,157 (44)	< 0.001
Non-Hispanic black	276 (17.9)	10,166 (23.0)	10,442 (22.8)	
Hispanic	265 (17.2)	12,298 (27.8)	12,563 (27.4)	
Other	59 (3.8)	2,599 (5.9)	2,658 (5.8)	
Obesity, n (%)				
Underweight	24 (1.6)	716 (1.6)	740 (1.6)	< 0.001
Normal	329 (21.3)	13,758 (31.1)	14,087 (30.7)	
Overweight	528 (34.3)	14,895 (33.6)	15,423 (33.7)	
Obese	629 (40.8)	14,535 (32.8)	15,164 (33.1)	
Unknown	31 (2.0)	375 (0.8)	406 (0.9)	
Poverty-income ratio, n (%)				
< 130%	568 (36.9)	12,340 (27.9)	12,908 (28.2)	< 0.001
130%-349%	543 (35.2)	15,775 (35.6)	16,318 (35.6)	
≥ 350%	315 (20.4)	12,692 (28.7)	13,007 (28.4)	
Unknown	115 (7.5)	3,472 (7.8)	3,587 (7.8)	
Education, n (%)				
< High school	622 (40.4)	12,414 (28.0)	13,036 (28.5)	< 0.001
High school	382 (24.8)	11,297 (25.5)	11,679 (25.5)	
> High school	531 (34.5)	20,473 (46.2)	21,004 (45.8)	
Unknown	6 (0.4)	95 (0.2)	101 (0.2)	
Physical activity, n (%)				
Inactive	366 (23.8)	11,148 (25.2)	11,514 (25.1)	< 0.001
Insufficiently active	383 (24.9)	16,576 (37.4)	16,959 (37.0)	
Active	790 (51.3)	16,541 (37.4)	17,331 (37.8)	
Unknown	2 (0.1)	14 (0.0)	16 (0.0)	
Alcohol consumption, n (%)				
0 drink/week	454 (29.5)	7,235 (16.3)	7,689 (16.8)	< 0.001
< 1 drink/week	316 (20.5)	11,252 (25.4)	11,568 (25.2)	
1–6 drinks/week	215 (14.0)	9,637 (21.8)	9,852 (21.5)	
≥ 7 drinks/week	166 (10.8)	5,800 (13.1)	5,966 (13.0)	
Unknown	390 (25.3)	10,355 (23.4)	10,745 (23.5)	
Smoking status, n (%)				
Past smoker	343 (22.3)	10,024 (22.6)	10,367 (22.6)	< 0.001
Current smoker	673 (43.7)	9,890 (22.3)	10,563 (23.1)	
Never	525 (34.1)	2,4345 (55)	24,870 (54.3)	
Unknown	0 (0.0)	20 (0.0)	20 (0.0)	
Hypertension, n (%)				
Yes	1,037 (67.3)	12,357 (27.9)	13,394 (29.2)	< 0.001
No	503 (32.6)	31,669 (71.5)	32,172 (70.2)	
Unknown	1 (0.1)	253 (0.6)	254 (0.6)	
Hypercholesterolemia, n (%)				
Yes	868 (56.3)	11,053 (25.0)	11,921 (26.0)	< 0.001
No	496 (32.2)	19,110 (43.2)	19,606 (42.8)	
Unknown	177 (11.5)	14,116 (31.9)	14,293 (31.2)	
Diabetes, n (%)				
Yes	409 (26.5)	3,637 (8.2)	4,046 (8.8)	< 0.001
No	1,089 (70.7)	40,028 (90.4)	41,117 (89.7)	
Unknown	43 (2.8)	614 (1.4)	657 (1.4)	

^a^Comparison between those with or without a prior diagnosis of MI.

^b^Representing the usual intake of nutrients.

*d* day, *IQR* Interquartile range, *MI* Myocardial infarction, *MUFA* Monounsaturated fatty acid, *n* number, *NA* Not applicable, *PUFA* Polyunsaturated fatty acid, *SD* Standard deviation, *SFA* Saturated fatty acid.

### Association of usual intake of fatty acids with heart disease mortality

This cohort was followed up for 532,722 person-years with a mean follow-up of 11.6 years. During the follow-up, 2,313 deaths from heart disease were recorded. Usual intake of SFAs and MUFAs was not associated with the risk of mortality from heart disease after adjustment for all the tested confounders (Table 2). In contrast, a 5% higher calorie intake from PUFAs was associated with a 9% lower multivariate-adjusted risk of heart disease mortality (HR, 0.91; 95% CI 0.83–1.00; *P* = 0.048; Table 2). Sub-analyses showed that this inverse association was present in those without a prior diagnosis of MI (HR, 0.89; 95% CI 0.80–0.99; *P* = 0.032) but not in those with the condition (HR, 0.94; 95% CI 0.75–1.16; *P* = 0.546; Table 2). The inverse association of PUFAs with heart disease mortality seemed mainly attributed to n6 fatty acids (Supplementary Table 3).

**Table 2:** A 5% higher calorie intake from usual intake of fatty acids and risk for heart disease mortality among 45,820 adults.

Models	SFA		MUFA		PUFA	
	HR (95% CI)	*P* value	HR (95% CI)	*P* value	HR (95% CI)	*P* value
Overall (N = 45,820)						
Model 1	1.19 (1.11–1.28)	< 0.001	1.13 (1.05–1.21)	0.002	0.81 (0.74–0.89)	< 0.001
Model 2	1.17 (1.08–1.26)	< 0.001	1.06 (0.99–1.14)	0.118	0.83 (0.75–0.92)	< 0.001
Model 3	1.00 (0.93–1.08)	0.986	0.93 (0.86–1.01)	0.074	0.89 (0.81–0.97)	0.012
Model 4	1.02 (0.94–1.10)	0.673	0.95 (0.88–1.03)	0.224	0.91 (0.83–1.00)	0.048
Participants with prior MI (N = 1,541)						
Model 1	1.12 (0.94–1.33)	0.198	1.10 (0.94–1.30)	0.245	0.94 (0.75–1.17)	0.571
Model 2	1.11 (0.93–1.33)	0.234	1.08 (0.91–1.27)	0.386	0.93 (0.75–1.17)	0.549
Model 3	1.01 (0.84–1.23)	0.892	0.95 (0.80–1.13)	0.590	0.95 (0.77–1.18)	0.649
Model 4	1.05 (0.86–1.27)	0.652	0.95 (0.80–1.13)	0.540	0.94 (0.75–1.16)	0.546
Participants without prior MI (N = 44,279)						
Model 1	1.21 (1.12–1.32)	< 0.001	1.12 (1.04–1.22)	0.005	0.79 (0.71–0.88)	< 0.001
Model 2	1.20 (1.10–1.30)	< 0.001	1.06 (0.97–1.15)	0.185	0.80 (0.72–0.90)	< 0.001
Model 3	1.03 (0.94–1.12)	0.572	0.94 (0.86–1.02)	0.121	0.86 (0.78–0.96)	0.007
Model 4	1.04 (0.95–1.13)	0.413	0.96 (0.88–1.05)	0.340	0.89 (0.80–0.99)	0.032

Model 1: adjusted for age; Model 2: adjusted for age, sex, and ethnicity; Model 3: adjusted for age, sex, ethnicity, obesity, poverty-income ratio, education, physical activity, alcohol consumption, smoking status, survey period, usual intake of protein (natural log transformed), and usual intake of carbohydrate (natural log transformed); Model 4: adjusted for all the factors in Model 3 plus systolic blood pressure (natural log transformed), total cholesterol (natural log transformed), and hemoglobin A_1c_ (natural log transformed).

*CI* Confidence interval, *HR* Hazard ratio, *MI* Myocardial infarction, *MUFA* Monounsaturated fatty acid, *PUFA* Polyunsaturated fatty acid, *SFA* Saturated fatty acid.

Similar results were obtained when the percentage of calories from fatty acids was treated as a categorical variable (*i.e*., quartile). In participants without a prior diagnosis of MI, people with PUFA intake in the highest quartile (mean percentage of calories from PUFAs in this quartile = 10.2%) had a 13% lower multivariate-adjusted risk of heart disease mortality (HR, 0.87; 95% CI 0.77–0.99; *P* = 0.029; Table 3) compared to those with an intake of PUFAs in the lowest quartile (mean percentage of calories from PUFAs in this quartile = 5.0%). However, in participants with a prior diagnosis of MI, quartiles of PUFA intake were not associated with heart disease mortality risk (Table 3).

**Table 3:** Quartiles of the percentage of calories from usual intake of fatty acids and risk for heart disease mortality among 45,820 adults.

Quartile	SFA				MUFA				PUFA			
	%Cal^a^	Gram^b^	HR^c^ (95% CI)	*P* value	%Cal^a^	Gram^b^	HR^c^ (95% CI)	*P* value	%Cal^a^	Gram^b^	HR^c^ (95% CI)	*P* value
Overall (N = 45,820)												
Q1	7.7%	16.0	Reference		9.1%	18.6	Reference		5.0%	10.9	Reference	
Q2	10.1%	22.1	1.00 (0.89–1.13)	0.963	11.5%	25.3	0.91 (0.80–1.03)	0.121	6.6%	14.7	0.98 (0.88–1.10)	0.737
Q3	11.7%	26.4	1.00 (0.88–1.13)	0.972	13.1%	29.7	1.04 (0.92–1.17)	0.540	7.9%	17.4	0.88 (0.78–0.99)	0.035
Q4	14.3%	32.1	1.06 (0.94–1.20)	0.313	15.8%	35.5	0.94 (0.83–1.06)	0.278	10.2%	21.7	0.91 (0.82–1.02)	0.115
Participants with prior MI (N = 1,541)												
Q1	7.7%	9.9	Reference		9.2%	16.4	Reference		5.1%	9.9	Reference	
Q2	10.0%	13.1	1.00 (0.76–1.32)	0.988	11.6%	22.9	1.04 (0.79–1.37)	0.767	6.7%	13.1	1.07 (0.81–1.40)	0.638
Q3	11.8%	15.9	1.17 (0.88–1.54)	0.279	13.2%	26.6	0.98 (0.75–1.29)	0.905	8.0%	15.9	0.87 (0.66–1.15)	0.317
Q4	14.3%	19.5	1.12 (0.85–1.49)	0.423	16.0%	31.7	0.99 (0.75–1.31)	0.927	10.3%	19.5	0.96 (0.74–1.24)	0.756
Participants without prior MI (N = 44,279)												
Q1	7.7%	16.0	Reference		9.1%	18.7	Reference		5.0%	10.9	Reference	
Q2	10.1%	22.2	1.01 (0.88–1.15)	0.935	11.5%	25.4	0.90 (0.78–1.04)	0.145	6.6%	14.8	0.95 (0.83–1.08)	0.409
Q3	11.7%	26.5	0.98 (0.85–1.13)	0.777	13.1%	29.9	1.05 (0.92–1.20)	0.504	7.9%	17.4	0.88 (0.78–1.01)	0.065
Q4	14.3%	32.3	1.10 (0.96–1.26)	0.175	15.7%	35.6	0.95 (0.83–1.09)	0.468	10.2%	21.8	0.87 (0.77–0.99)	0.029

^a^Mean percentage of calories from fatty acids in each quartile.

^b^Mean amount (grams) of fatty acid intake in each quartile.

^c^Adjusted for age, sex, ethnicity, obesity, poverty-income ratio, education, physical activity, alcohol consumption, smoking status, survey period, usual intake of protein (natural log transformed), usual intake of carbohydrate (natural log transformed), systolic blood pressure (natural log transformed), total cholesterol (natural log transformed), and hemoglobin A1c (natural log transformed).

*CI* Confidence interval, *HR* Hazard ratio, *MI* Myocardial infarction, *MUFA* Monounsaturated fatty acid, *PUFA* Polyunsaturated fatty acid, *Q* Quartile, *SFA* Saturated fatty acid.

When the amount (measured in g) of usual intake of fatty acids was used, a 1-natural-log higher PUFA intake (*e.g*., 27 g versus 10 g per day) was associated with an 11% (HR, 0.89; 95% CI 0.79–1.00; *P* = 0.041) lower multivariate-adjusted risk of heart disease mortality. Similarly, this association was present in those without a prior diagnosis of MI (HR, 0.87; 95% CI 0.77–0.99; *P* = 0.035; Supplementary Table 4) but not in those with the condition (HR, 0.93; 95% CI 0.70–1.22; *P* = 0.577).

### Sensitivity analysis

Similar results were obtained when imputed data were not used, via (1) using categorical variables (Supplementary Tables 5–6) or (2) excluding those who had missing data (Supplementary Tables 7–8). Similar results were also obtained when fatty acid intake was expressed as mean intake from the two survey days (Supplementary Table 9), when the analysis was further adjusted for C-reactive protein (Supplementary Table 10) or total energy intake (Supplementary Table 11) or the use of aspirin and statin (Supplementary Table 12), when those who had a mean total caloric intake of < 500 or > 3,500 kcal/day were excluded (Supplementary Table 13), or when total cholesterol was replaced with LDL cholesterol for adjustment (Supplementary Table 14).

### Association of fatty acid intake with lipid profile in 44,279 adults without a prior diagnosis of MI

**Figure 1:**
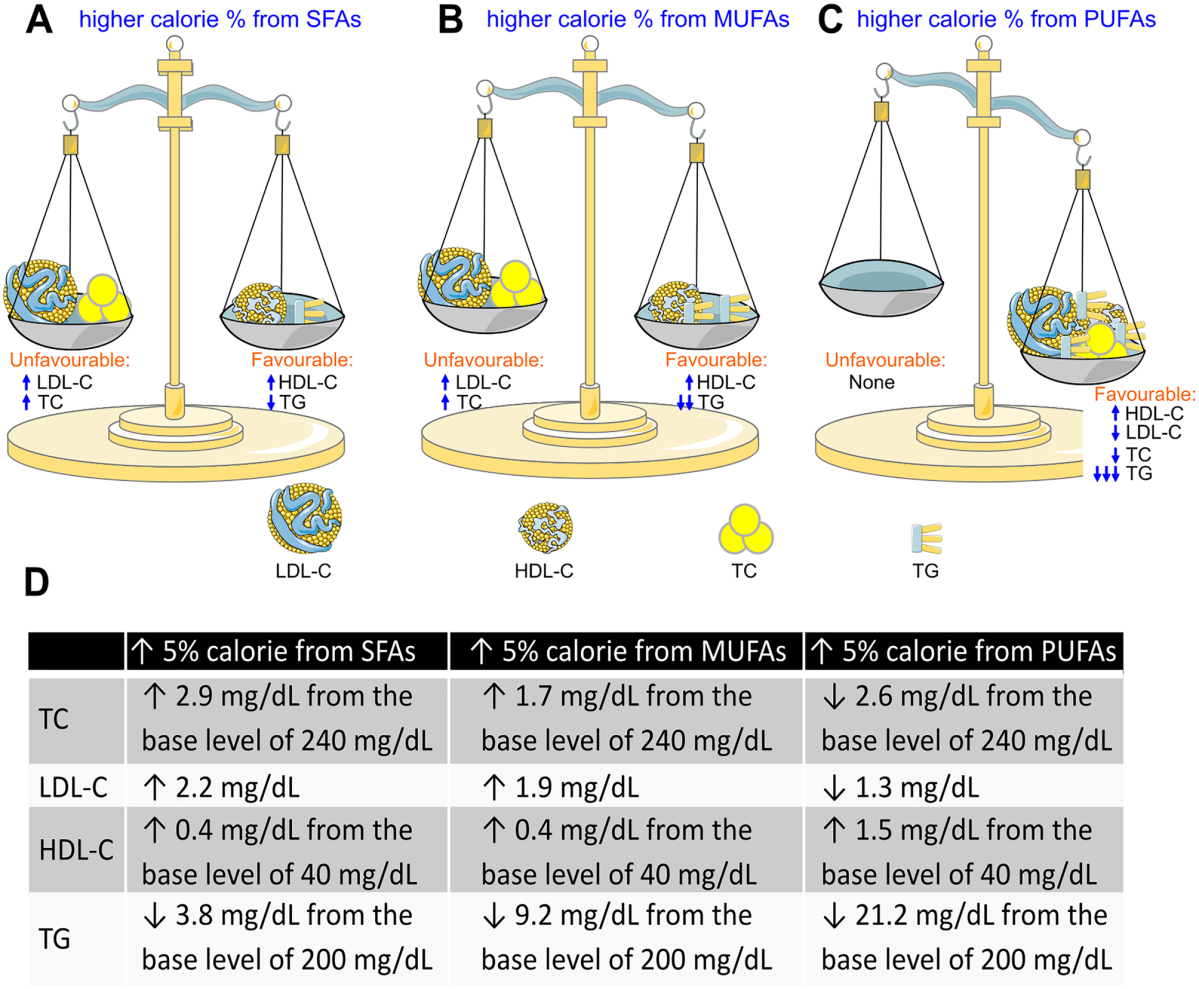
Lipid profile associated with usual intake of fatty acids in 44,279 adults without a prior diagnosis of myocardial infarction. (**A-B)**, Higher usual intake of saturated fatty acids (SFAs, **A**) and monounsaturated fatty acids (MUFAs, **B**) was associated with higher low-density lipoprotein cholesterol (LDL-C), higher total cholesterol (TC), higher high-density lipoprotein cholesterol (HDL-C) and lower triglyceride (TG). (**C**), Higher usual intake of polyunsaturated fatty acids (PUFAs) was associated with higher HDL-C, lower LDL-C, lower TC, and lower TG. (**D**) The extent of change in lipid profile associated with a 5% higher calorie intake from fatty acids. This figure was partly generated using Servier Medical Art, provided by Servier, licensed under a Creative Commons Attribution 3.0 unported license.

Dietary intake of SFAs and MUFAs was associated with both favourable and unfavourable lipid profiles: higher intake of SFAs and MUFAs was associated with slightly but significantly higher levels of total cholesterol, LDL cholesterol, and HDL cholesterol, and associated with a slightly but significantly lower level of triglyceride (Fig. 1).

In contrast, higher intake of PUFAs was associated with favourable profiles across all lipid species, *i.e.*, lower total cholesterol, lower LDL cholesterol, lower triglyceride, and higher HDL cholesterol (Fig. 1). However, after further adjustment for all these lipid variables, the inverse association between PUFAs and risk of heart disease mortality remained significant in those without a prior diagnosis of MI (Table 4).

**Table 4:** A 5% higher calorie intake from usual intake of fatty acids and heart disease mortality risk with further adjustment for LDL cholesterol, HDL cholesterol, and triglyceride among 44,279 adults without a prior diagnosis of MI.

Models	SFA		MUFA		PUFA	
	HR (95% CI)	*P* value	HR (95% CI)	*P* value	HR (95% CI)	*P* value
Model 1	1.04 (0.95–1.13)	0.413	0.96 (0.88–1.04)	0.340	0.89 (0.80–0.99)	0.032
Model 2	1.04 (0.95–1.13)	0.385	0.96 (0.88–1.05)	0.351	0.89 (0.80–0.99)	0.035

Model 1: adjusted for age, sex, ethnicity, obesity, poverty-income ratio, education, physical activity, alcohol consumption, smoking status, survey period, usual intake of protein (natural log transformed), usual intake of carbohydrate (natural log transformed), systolic blood pressure (natural log transformed), total cholesterol (natural log transformed), and hemoglobin A_1c_ (natural log transformed).

Model 2: adjusted for all the factors in Model 1 plus LDL cholesterol, HDL cholesterol (natural log transformed), and triglyceride (natural log transformed).

*CI* Confidence interval, *HDL* High-density lipoprotein, *HR* Hazard ratio, *LDL* Low-density lipoprotein, *MI* Myocardial infarction, *MUFA* Monounsaturated fatty acid, *PUFA* Polyunsaturated fatty acid, *SFA* Saturated fatty acid.

## Discussion

Using a large representative cohort of US adults (N = 45,820) with an extensive follow-up period of 11.6 years (mean), this study revealed that percentage of calories from usual intake of SFAs and MUFAs was not associated with adjusted risk of heart disease mortality. By contrast, percentage of calories from usual intake of PUFAs was inversely associated with heart disease mortality risk and this inverse association was only present in those without a prior diagnosis of MI.

Similar findings were obtained regardless of whether the amount (g) of usual intake of fatty acids or percentage of calories from the mean intake of fatty acids were used for analysis. Similar findings were also obtained when the usual intake of fatty acids was treated as categorical variables (quartiles). In addition, the findings remained similar when the missing data were not imputed, either by excluding those small number of participants with missing data from the analysis or by using categorical variables (including an unknown category), or when the analysis was further adjusted for body mass index and total energy intake.

The finding that intake of SFAs was not associated with heart disease mortality might, to a certain extent, disagree with the American Heart Association’s position that SFAs are a major risk factor for coronary heart disease (CHD) events^4,5^. However, whether SFAs were associated with non-fatal CHD events in our study was unknown. Our finding is consistent with a number of recent meta-analyses which showed that reductions of saturated fat intake do not protect against heart disease mortality^22,23^. The high intake of SFAs could increase total cholesterol; however, the magnitude of this increase was limited in this general cohort: a 5% higher calorie intake from SFAs was only associated with a 2.9 mg/dL higher level of total cholesterol including a 2.2 mg/dL higher level of LDL cholesterol. The small impact of SFAs on total cholesterol was consistent with a previous study with a smaller sample size (N = 99)^25^ which showed that intake of total animal fat (mainly SFAs) was not associated with plasma cholesterol levels. On the other hand, a 5% higher calorie intake from SFAs was associated with a 0.4 mg/dL higher level of HDL cholesterol, which may be expected to protect against heart disease. This observation was consistent with the study from Ekanayaka et al^26^ which showed that consumption of coconut milk (primarily consisting of SFAs) was associated with an increase in HDL cholesterol in Sri Lankans. Therefore, the lipid profile associated with higher SFA intake seemed balanced (Fig. 1).

This study also showed that intake of MUFAs was not associated with mortality from heart disease. This finding was consistent with a number of meta-analyses of prospective cohort studies which showed that dietary MUFA intake was not associated with coronary events and heart disease mortality^24,27,28^.

The finding that PUFAs were inversely associated with heart disease mortality was consistent with some RCTs^5,8,29^. It has been shown that increased PUFA intake reduces the severity of stenoses in the coronary arteries^19^. The effects of PUFAs are generally thought to be mediated by lowering total cholesterol, LDL cholesterol and triglyceride^5^; consistent with this, our results showed that higher intake of PUFAs was associated with lower levels of total cholesterol, LDL cholesterol and triglyceride in the plasma. However, Dayton et al. suggested that the protective effect of PUFAs against cardiovascular events may be not mediated by total cholesterol^9^. That trial showed that higher intake of PUFAs decreased cardiovascular events in younger (< 65.5 years) but not older participants; however, the extent of the decrease in the serum cholesterol associated with higher intake of PUFAs was smaller in younger (11.7%) compared with older participants (13.7%)^9^. Similarly, the British Medical Research Council Soy Oil trial^13^ showed that the relapse of angina and MI was not associated with cholesterol levels at baseline nor with a change in cholesterol determined during the clinical trial. Our results showed that PUFAs remained inversely associated with heart disease mortality after adjustment for total cholesterol, HDL cholesterol, LDL cholesterol and triglyceride, suggesting that PUFAs may, at least in part, work through alternative mechanisms. Indeed, animal studies suggest that PUFAs may directly affect cardiomyocytes via their receptors *e.g*., free fatty acid receptor 4 (FFAR4)^30^. However, whether this is the case in humans remains to be investigated. In addition, PUFAs could work through anti-oxidative effects^31^. Regardless of the exact mechanism, our results suggest that increasing PUFA intake may be an effective preventive strategy to reduce heart disease mortality in the general population. In addition, our study suggested that the inverse association of dietary PUFAs with heart disease mortality was mainly attributed to n6 fatty acids. This result is consistent with a recent analysis of 30 cohort studies which showed that higher circulating and tissue levels of linoleic acid (the major dietary n6 fatty acid) were associated with a lower risk of major cardiovascular events and mortality^32^.

This study found that the inverse association between PUFA and heart disease mortality was present in those without pre-existing MI, but not in those with the condition. The reason for this is not clear. It is possible that PUFAs inhibit the progression of atherosclerosis but do not improve the stability of the plaque. In those with MI, *i.e.,* well-established coronary atherosclerosis, higher PUFA can still inhibit the progression of atherosclerosis in the coronary arteries^19^. However, MI has been found to frequently develop from previous non-severe lesions^33^, and the severity of the coronary stenosis on angiogram before MI is not associated with the time from this initial angiogram to acute MI^34^. These studies suggested MI and death in those with established coronary atherosclerosis may depend more on the stability of the plaque rather than overall plaque burden. Our study, together with some RCTs showing that higher PUFAs were not associated with protective effects against re-infarction^13,17^, suggests that PUFAs may not improve the stability of established atherosclerotic plaques. In fact, the Sydney Diet Heart Study^20^ and the Rose Corn Oil trial^12^ suggested that PUFAs may even reduce the stability of established atherosclerosis as the intervention showed worsened heart disease survival. Overall, our results, together with other reports^12,13,17,20^, suggest that increasing PUFA intake might not reduce heart disease mortality in those who are suffering from coronary heart disease in the general public.

The mean percentage of calories from PUFAs in our study was 7.4%, which was higher than the mean of 5.7% in the control groups of the available RCTs (Supplementary Table 2). This is expected as all these RCTs were conducted before 1992: a period when the American Heart Association’s public health messages might be expected to have increased the intake of PUFAs in the US general public^4,5^. Of note, the mean percentage of calories from PUFAs of 17.2% in the intervention groups of the published RCTs is much higher than the mean of 10.2% in the highest quartiles in the general public in our study. The percentage of calories from PUFAs in the Medical Research Council Soy Oil Trial^13^ was 31.3% (85 g soybean oil per day) and at least 43 g of oil per day was reported to be drunk with fruit juice^13^. Therefore, the very high doses of PUFAs in these RCTs do not likely mimic the levels that arise through regular dietary intake in the general public.

The high doses of PUFAs in those RCTs are reflected by a large drop in cholesterol in the blood (mean drop, 30.6 mg/dL; Supplementary Table 2). However, coronary heart disease events were not associated with the extent of decrease in cholesterol during the trial^13^; those high doses of PUFAs could even worsen coronary heart disease outcomes^12,20^; and the Minnesota Coronary Survey^21^ showed that each 30 mg/dL reduction in serum cholesterol during the trial was associated with a 22% higher risk of death. Therefore, these results suggest that the high doses of dietary PUFAs used in many RCTs might have toxic effects. Consequently, some meta-analysis reports showed that increasing dietary intake of PUFAs in RCTs failed to show protection against heart disease mortality^20,21^.

**Figure 2:**
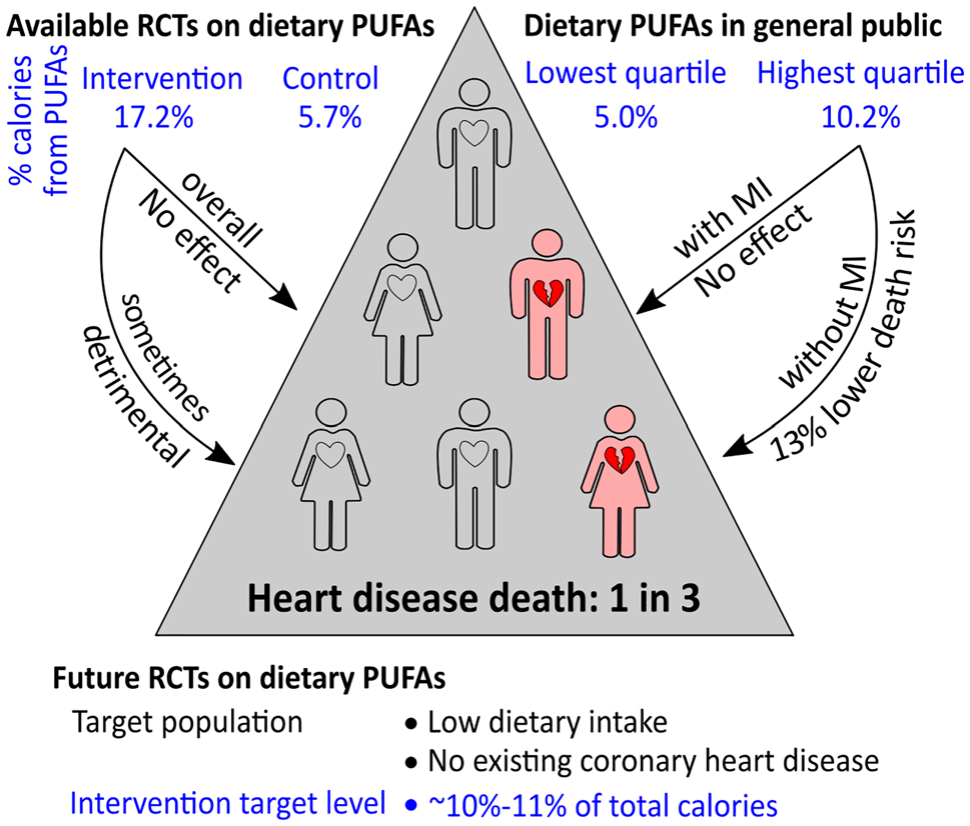
Suggestions for future randomised controlled trials (RCTs) investigating dietary polyunsaturated fatty acids (PUFAs) on heart disease mortality. Available RCTs suggest that dietary PUFAs may not protect against heart disease mortality overall. However, those trials used high doses of dietary PUFAs, which do not likely mimic diet in the general public, and the high doses of dietary PUFAs used in many RCTs might have toxic effects. Some meta-analysis reports showed that increasing dietary intake of PUFAs in RCTs failed to show protection against heart disease mortality. Our results showed that, in people from the general public who did not have a prior diagnosis of myocardial infarction (MI), those with PUFA intake in the highest quartile had a 13% lower heart disease mortality risk compared to those in the lowest quartile. Future RCTs could target those without established coronary heart disease and with low dietary PUFA intake, with the intervention to increase PUFA intake to more modest levels (*e.g*., 10–11%, equivalent to the mean level in the highest quartile of our study).

Our study suggests that a new RCT may be warranted to test the effect of PUFAs on heart disease events and mortality in at-risk individuals with low intake of PUFAs, in which the intervention increases PUFA to more modest levels (*e.g*., 10–11%, equivalent to the mean level in the highest quartile of our study) (Fig. 2). In addition, such an RCT would likely focus on those without established coronary heart disease to test the primary preventive effect of PUFAs. Positive findings from such a trial would have profound public health implications, given that heart disease mortality accounts for about one third of all deaths^1^.

It has been well known that food choice affects health. For example, the western diet increases cardiovascular mortality risk whereas healthy foods (*e.g*., fish and whole grain) could decrease it^35^. The major food sources of the fatty acids in this study were listed in Supplementary Table 15). Overall, the diet with high PUFA contained more healthy-food items such as fish, sunflower seed, flax seed, and nuts. Whether the food sources of fatty acids affect the associations between fatty acids and heart disease mortality in our study is unclear. Of note, French fries represented a major source of PUFAs (Supplementary Table 15). French fries are generally perceived as less healthy; however, two recent studies showed that French fries were not associated with cardiovascular disease mortality^36,37^.

### Strengths and limitations

This study has several strengths. First, it has a large sample size (N = 45,820). Second, these participants were selected to represent noninstitutionalised US civilian persons. Therefore, the findings and conclusions of this study could be extrapolated to the general noninstitutionalised US adult population. Third, the analyses were adjusted for a number of common confounding factors. Several limitations are also identified in this study. First, mortality outcomes were ascertained by linkage to the NDI records with a probabilistic match, which may lead to misclassification. However, a prior validation study showed high accuracy of the matching method (98.5%)^38^. Second, the findings of this study exclude those living in institutions such as nursing homes and prisons. Third, dietary and lifestyle changes over time were not assessed which may lead to misclassification. Nevertheless, in epidemiological analysis, this misclassification tends to result in an underestimate rather than an overestimate of risk because of the effect of regression dilution bias^39^.

In conclusion, this study suggests that higher intake of PUFAs, but not SFAs nor MUFAs, is associated with a lower risk of heart disease mortality. The inverse association between PUFAs and heart disease mortality was only present in those without a prior diagnosis of MI. Our results suggest that increasing PUFA intake may be an effective primary preventive strategy to reduce heart disease mortality in the general public, and thus a future RCT aimed at increasing PUFA intake to 10–11% of the total calorie intake may be warranted to test this hypothesis (Fig. 2).

## Methods

### Study participants

NHANES is a continuous, nationally representative survey consisting of about 5,000 non-institutionalised persons each year^40^. A total of 54,960 adults aged ≥ 20 years attended the NHANES examination at the Mobile Examination Center from 1988 to 2014 and had dietary intake data which were reliable and met the minimum criteria (judged by National Center for Health Statistics staff). The following participants were excluded progressively: those without a follow-up time or with a follow-up time of 0 month (N = 76), those without MI status (N = 270), those with a mean total caloric intake of 0 kcal (N = 2), and those who died of a cause other than heart disease (N = 8,792). Therefore, a total of 45,820 participants were included in the final analysis (Supplementary Figure 1).

### Ethical considerations

The study was conducted following the ethical standards laid down in the Declaration of Helsinki. It was approved by the NHANES Institutional Review Board (currently known as National Center for Health Statistics Research Ethics Review Board). All participants provided written informed consent. All participant records were anonymised before being accessed by the authors^41^.

### Dietary intake of fatty acids

Dietary intake data were obtained from two automated multiple-pass 24-h dietary recall interviews. The first interview (Day 1) was collected in person in the Mobile Examination Center and the second (Day 2) was collected by telephone 3 to 10 days later^42^. Dietary intake of fatty acids (SFAs, MUFAs, and PUFAs) on each dietary interview day was directly obtained from the NHANES datasets, and these two sets of data were used to determine daily usual intake or mean intake for further analysis.

Daily usual intake of fatty acids for each participant was determined as previously reported^43^ using the MSM program^44^ with survey day (Day 1 or Day 2) and a weekend day flag (Friday/Saturday/Sunday versus others) as covariates^45^. The MSM program, a web-based statistics package for estimating usual dietary intake, was accessed through the following address: https://msm.dife.de. Daily usual intake of fatty acids was expressed as either the percentage of calories from fatty acids or grams of fatty acids. The former was calculated using the following formula^46^: percentage of calories from fatty acids = (grams of fatty acids X 9 / total calorie intake in kcal) X 100.

In the sensitivity analysis, the mean fatty acid intake from the two survey days^47^ was used instead of usual intake.

### Mortality from heart disease

Data on mortality from heart disease (I00-I09, I11, I13, I20-I51) were retrieved from NHANES-linked mortality files^41^. To evaluate mortality status and the cause of death, the National Center for Health Statistics conducted probabilistic matching^48^ to link the NHANES data with death certificate records from the National Death Index (NDI) records. The NHANES-linked mortality files used the Underlying Cause of Death 113 (UCOD_113) code to recode all deaths according to the International Classification of Diseases, 9th Revision (ICD-9) or the International Classification of Diseases, 10th Revision (ICD–10) for underlying cause of death. Follow-up time was the duration from the time when the participant was examined at the Mobile Examination Center until death, or until the end of follow-up (December 31, 2015), whichever occurred first^41^.

### Covariates

Confounding covariates were similar to previous reports^49,50^. They included age (continuous), sex (male or female), ethnicity (Hispanic, non-Hispanic white, non-Hispanic black, or other), obesity (underweight, normal, overweight, obese, or unknown), education (< high school, high school, > high school, or unknown), poverty-income ratio (< 130%, 130%-349%, ≥ 350%, or unknown), and survey periods (1988–1991, 1991–1994, 1999–2000, 2001–2002, 2003–2004, 2005–2006, 2007–2008, 2009–2010, 2011–2012, or 2013–2014). Lifestyle confounders included physical activity (inactive, insufficiently active, or active), alcohol consumption (never, < 1 drink per week, 1–6 drinks per week, ≥ 7 drinks per week, or unknown), and smoking status (past smoker, current smoker, non-smoker, or unknown). Clinical confounders included systolic blood pressure (continuous), total cholesterol (continuous), and hemoglobin A_1c_ (continuous); self-reported physician diagnoses (yes, no, or unknown) of hypertension, hypercholesterolemia, and diabetes were used in the sensitivity analysis. In addition, the usual intake of protein and carbohydrate (continuous) and plasma levels of low-density lipoprotein (LDL) cholesterol (continuous), high-density lipoprotein (HDL) cholesterol (continuous), and triglyceride (continuous) were also adjusted in the analysis.

### Statistical analyses

Data were presented as mean and standard deviation for normally distributed continuous variables, or median and interquartile range for non-normally distributed continuous variables, or number and percentage for categorical variables^51^. Differences in age between two groups were analysed using Student’s T-test, and differences in usual intake of fatty acids, protein and carbohydrate between two groups were analysed using the Mann–Whitney U test. Differences among categorical variables were analysed using Pearson’s Chi-square test. Cox proportional hazards models^40,52^ were used to calculate hazard ratios (HRs) and 95% confidence intervals (CIs) of usual intake of fatty acids for mortality from heart disease. The associations between the percentage of calories from fatty acids and plasma lipids were analysed by simple linear regression. Sub-analyses were conducted in sub-cohorts of participants stratified by prior diagnosis of MI. We chose to use a 5% difference in fatty acid intake in the analyses because the difference in the percentage of calorie intake from PUFAs between those in the top and bottom quartiles was about 5%.

In the main analyses, imputed values for systolic blood pressure, total cholesterol, and hemoglobin A_1c_ were used. Among 45,820 participants, 3,260 participants had missing values in either systolic blood pressure, total cholesterol, or hemoglobin A_1c_. These missing data were imputed through a multiple imputation approach using chained equations, with 20 imputed data sets being created^48^. Little’s test showed that the missing data were not missing completely at random (*P* < 0.001).

In the sub-analysis, PUFAs were further classified as n3 and n6 fatty acids as previously described^53^. In brief, n3 fatty acids were a sum of linolenic acid (18:3), stearidonic acid (18:4), eicosapentaenoic acid (20:5, EPA), docosapentaenoic acid (22:5, DPA), and docosahexaenoic acid (22:6, DHA), and n6 fatty acids were a sum of linoleic acid (18:2) and arachidonic acid (20:4).

In the sensitivity analysis, imputed data were not used, via (1) using categorical variables (*i.e*., hypertension, hypercholesterolemia, and diabetes, with an unknown category) or (2) excluding those 3,260 participants who had missing data from the analysis. In addition, sensitivity analyses were conducted by further adjustment for (1) C-reactive protein, (2) total energy intake, and (3) the use of aspirin and statin. Sensitivity analyses were also conducted by excluding those participants who had a mean total caloric intake of < 500 kcal or > 3500 kcal/day, or replacing total cholesterol with LDL cholesterol for adjustment*.*

The null hypothesis was rejected for two-sided *P* values of < 0.05. All analyses were performed using SPSS version 27.0 (IBM SPSS Statistics for Windows, Armonk, NY, IBM Corporation).

## Supplementary Information

Supplementary Information.

## Data Availability

All data in the current analysis are publicly available on the NHANES website.

## References

[1] Centers for Disease Control and Prevention, National Center for Health S. About Multiple Cause of Death, 1999–2020. CDC WONDER Online Database website. Atlanta, GA: Centers for disease control and prevention. Availalble from https://wonder.cdc.gov/mcd-icd10.html. (2022).

[2] Centers for Disease Control and Prevention. Heart Disease Facts. Available from https://www.cdc.gov/heartdisease/facts.htm.

[3] Tsao CW (2022). Heart disease and stroke statistics-2022 update: A report from the american heart association. Circulation.

[4] Page IH (1961). Dietary fat and its relation to heart attacks and strokes. Circulation.

[5] Sacks FM (2017). Dietary fats and cardiovascular disease: A presidential advisory from the American heart association. Circulation.

[6] Haring B, von Ballmoos MC, Appel LJ, Sacks FM (2014). Healthy dietary interventions and lipoprotein (a) plasma levels: Results from the omni heart trial. PLoS ONE.

[7] Root MM, Dawson HR (2013). DASH-like diets high in protein or monounsaturated fats improve metabolic syndrome and calculated vascular risk. Int. J. Vitam. Nutr. Res..

[8] Leren P (1968). The effect of plasma-cholesterol-lowering diet in male survivors of myocardial infarction. A controlled clinical trial. Bull. N. Y. Acad. Med..

[9] Dayton, S., Pearce, M. L., Hashimoto, S., Dixon, W. J. & Tomiyasu, U. A controlled clinical trial of a diet high in unsaturated fat in preventing complications of atherosclerosis. *Circulation* **40**, II-1-II-63 (1969).

[10] Pearce ML, Dayton S (1971). Incidence of cancer in men on a diet high in polyunsaturated fat. Lancet.

[11] Turpeinen O (1979). Dietary prevention of coronary heart disease: The finnish mental hospital study. Int. J. Epidemiol..

[12] Rose G, Thomson W, Williams R (1965). Corn oil in treatment of ischaemic heart disease. BMJ.

[13] Morris JN (1968). Controlled trial of soya-bean oil in myocardial infarction. Lancet.

[14] Woodhill JM, Palmer AJ, Leelarthaepin B, McGilchrist C, Blacket RB (1978). Low fat, low cholesterol diet in secondary prevention of coronary heart disease. Adv. Exp. Med. Biol..

[15] Houtsmuller AJ, van Hal-Ferwerda J, Zahn KJ, Henkes HE (1981). Favorable influences of linoleic acid on the progression of diabetic micro- and macroangiopathy in adult onset diabetes mellitus. Prog. Lipid Res..

[16] Miettinen M (1983). Dietary prevention of coronary heart disease in women: The finnish mental hospital study. Int. J. Epidemiol..

[17] Burr ML (1989). Effects of changes in fat, fish, and fibre intakes on death and myocardial reinfarction: Diet and reinfarction trial (DART). Lancet.

[18] Frantz I (1989). Test of effect of lipid lowering by diet on cardiovascular risk. The minnesota coronary survey. Arteriosclerosis.

[19] Watts G (1992). Effects on coronary artery disease of lipid-lowering diet, or diet plus cholestyramine, in the st thomas' atherosclerosis regression study (STARS). Lancet.

[20] Ramsden CE (2013). Use of dietary linoleic acid for secondary prevention of coronary heart disease and death: Evaluation of recovered data from the sydney diet heart study and updated meta-analysis. BMJ.

[21] Ramsden CE (2016). Re-evaluation of the traditional diet-heart hypothesis: analysis of recovered data from minnesota coronary experiment (1968–73). BMJ.

[22] Hooper L, Martin N, Abdelhamid A, Davey Smith G (2015). Reduction in saturated fat intake for cardiovascular disease. Cochrane Database Syst. Rev..

[23] Hooper L (2020). Reduction in saturated fat intake for cardiovascular disease. Cochrane Database Syst. Rev..

[24] Chowdhury R (2014). Association of dietary, circulating, and supplement fatty acids with coronary risk: A systematic review and meta-analysis. Ann. Intern. Med..

[25] Morris JN, Marr JW, Heady JA, Mills GL, Pilkington TR (1963). Diet and plasma cholesterol in 99 bank men. Br. Med. J..

[26] Ekanayaka RA, Ekanayaka NK, Perera B, De Silva PG (2013). Impact of a traditional dietary supplement with coconut milk and soya milk on the lipid profile in normal free living subjects. J. Nutr. Metab..

[27] Skeaff CM, Miller J (2009). Dietary fat and coronary heart disease: Summary of evidence from prospective cohort and randomised controlled trials. Ann. Nutr. Metab..

[28] Jakobsen MU (2009). Major types of dietary fat and risk of coronary heart disease: A pooled analysis of 11 cohort studies. Am. J. Clin. Nutr..

[29] Leren P (1970). The oslo diet-heart study. Eleven-year report. Circulation.

[30] O'Connell TD, Block RC, Huang SP, Shearer GC (2017). ω3-Polyunsaturated fatty acids for heart failure: Effects of dose on efficacy and novel signaling through free fatty acid receptor 4. J. Mol. Cell. Cardiol..

[31] Stocker R, Keaney JF (2004). Role of oxidative modifications in atherosclerosis. Physiol. Rev..

[32] Marklund M (2019). Biomarkers of dietary omega-6 fatty acids and incident cardiovascular disease and mortality. Circulation.

[33] Ambrose JA (1988). Angiographic progression of coronary artery disease and the development of myocardial infarction. J. Am. Coll. Cardiol..

[34] Hackett D, Verwilghen J, Davies G, Maseri A (1989). Coronary stenoses before and after acute myocardial infarction. Am. J. Cardiol..

[35] Heidemann C (2008). Dietary patterns and risk of mortality from cardiovascular disease, cancer, and all causes in a prospective cohort of women. Circulation.

[36] Larsson SC, Wolk A (2016). Potato consumption and risk of cardiovascular disease: 2 prospective cohort studies. Am. J. Clin. Nutr..

[37] Cruijsen E, Indyk IM, Simon AWE, Busstra MC, Geleijnse JM (2021). Potato consumption and risk of cardiovascular mortality and type 2 diabetes after myocardial infarction: A prospective analysis in the alpha omega cohort. Front. Nutr..

[38] Menke A, Muntner P, Batuman V, Silbergeld EK, Guallar E (2006). Blood lead below 0.48 micromol/L (10 microg/dL) and mortality among US adults. Circulation.

[39] MacMahon S (1990). Blood pressure, stroke, and coronary heart disease. Part 1, Prolonged differences in blood pressure: Prospective observational studies corrected for the regression dilution bias. Lancet.

[40] Wang Y (2022). Stage 1 hypertension and risk of cardiovascular disease mortality in United States adults with or without diabetes. J. Hypertens..

[41] Wang Y (2022). Fasting triglycerides are positively associated with cardiovascular mortality risk in people with diabetes. Cardiovasc. Res..

[42] Wang Y (2021). Tree nut consumption is associated with higher sex hormone-binding globulin levels in premenopausal US women. Nutr. Res..

[43] Wang Y, Fang Y (2022). Tree nut consumption is associated with a lower risk of hyperestrogenism in men. Nutr. Res..

[44] Harttig U, Haubrock J, Knüppel S, Boeing H (2011). The MSM program: Web-based statistics package for estimating usual dietary intake using the multiple source method. Eur. J. Clin. Nutr..

[45] O'Neil CE, Fulgoni VL, Nicklas TA (2015). Tree nut consumption is associated with better adiposity measures and cardiovascular and metabolic syndrome health risk factors in US Adults: NHANES 2005–2010. Nutr. J..

[46] Dehghan M (2017). Associations of fats and carbohydrate intake with cardiovascular disease and mortality in 18 countries from five continents (PURE): A prospective cohort study. Lancet.

[47] Austin GL, Ogden LG, Hill JO (2011). Trends in carbohydrate, fat, and protein intakes and association with energy intake in normal-weight, overweight, and obese individuals: 1971–2006. Am. J. Clin. Nutr..

[48] Wang Y, Fang Y, Sobey CG, Drummond GR (2023). Prior cancer diagnosis and mortality profile in US adults. Am. J. Med. Sci..

[49] Fang Y, Wang Y (2022). Fasting status modifies the association between triglyceride and all-cause mortality: A cohort study. Health Sci Rep.

[50] Wang Y (2021). Higher fasting triglyceride predicts higher risks of diabetes mortality in US adults. Lipids Health Dis..

[51] Wang Y, Charchar FJ (2021). Establishment of sex difference in circulating uric acid is associated with higher testosterone and lower sex hormone-binding globulin in adolescent boys. Sci. Rep..

[52] Wang Y, Fang Y (2022). Late non-fasting plasma glucose predicts cardiovascular mortality independent of hemoglobin A1c. Sci. Rep..

[53] Chen J, Sun B, Zhang D (2019). Association of dietary n3 and n6 fatty acids intake with hypertension: NHANES 2007–2014. Nutrients.

