# Supplementary material for: Dietary fatty acids and mortality risk from heart disease in US adults: an analysis based on NHANES
Source: Sci Rep. 2023 Jan 28;13:1614. doi: 10.1038/s41598-023-28738-2 (PMC9884296; doi:10.1038/s41598-023-28738-2)
Supplement: Supplementary file 1 — Supplementary Information. [file 41598_2023_28738_MOESM1_ESM.pdf]

Supplementary Information

Title: Dietary fatty acids and mortality risk from heart disease in US adults: An analysis based on NHANES

Authors: Yutang Wang, Yan Fang, Paul K. Witting, Fadi J. Charchar, Christopher G. Sobey, Grant R. Drummond, Jonathan Golledge

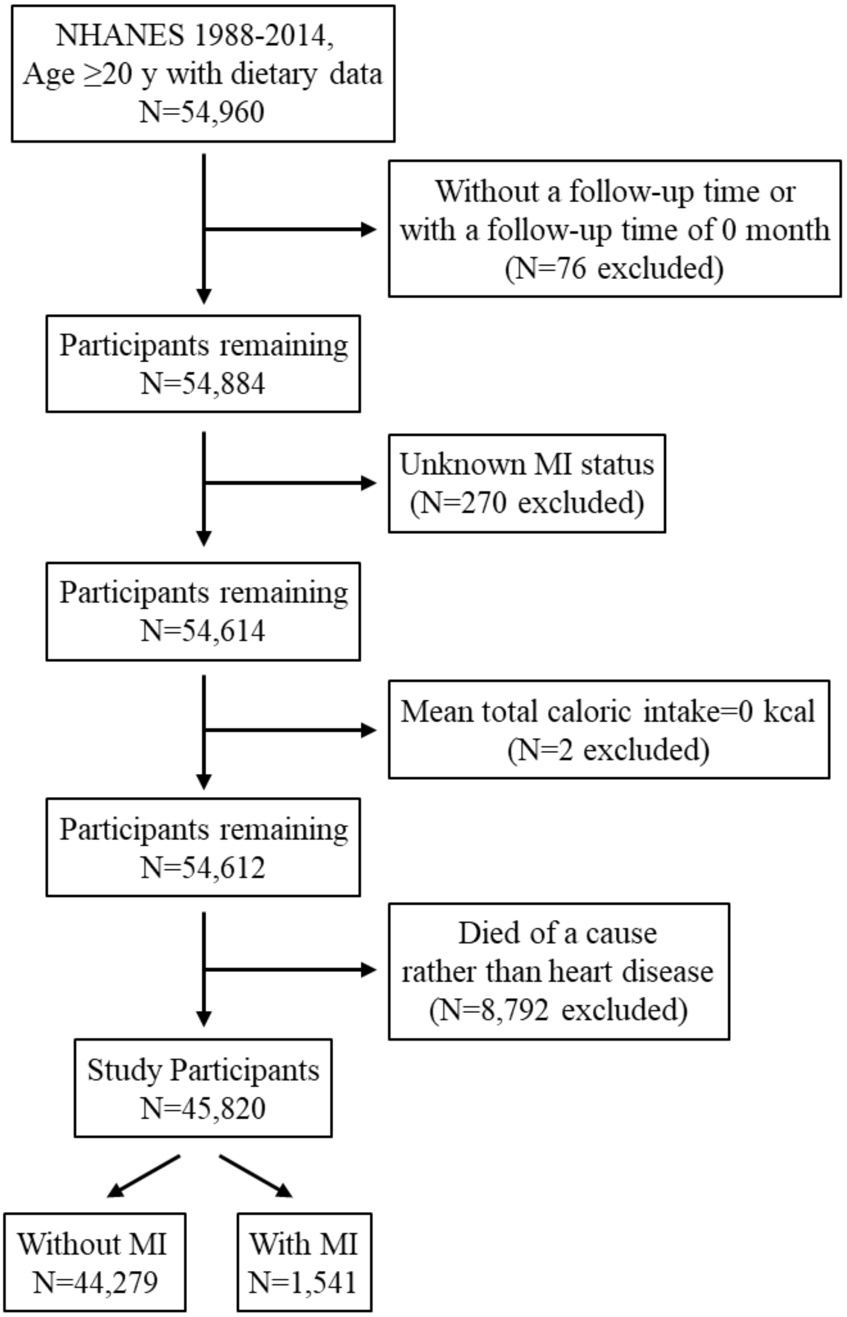

Supplementary Figure 1. Flow diagram of the study participants. MI, myocardial infarction; NHANES, National Health and Nutrition Examination Surveys.

Supplementary Table 1. Eleven randomised controlled trials investigating the effect of dietary polyunsaturated unsaturated fatty acids (PUFAs) on heart disease

Trial number	Trial name	Pub year	Participants	PUFAs beneficial?	Main results
1	Rose Corn Oil Trial[1]	1965	With CHD	No. Detrimental	% patients remaining alive and free of reinfarction were 75 % vs 52 % in the control vs treatment groups
2	MRC Soy Oil Trial[2]	1968	With MI	No	No effect on relapse
3	Oslo Diet-Heart Study[3]	1968	With MI	Yes	29% (*P*=0.011) decrease in recurrent MI and new cases of angina or sudden death
4	Los Angeles Veterans[4]	1969	Veterans	Maybe	20% fewer MI or sudden death, but *P*>0.05
4	Further report on Los Angeles Veterans[5]	1971	Veterans	Yes	↓ fatal atherosclerotic events
5	Sydney Diet Heart Study[6]	1978	With CHD	No. Detrimental	Intervention had a slightly but significantly worse survival.
6	The Finnish Mental Hospital Men Study[7]	1979	Hospital male patients	Yes	↓ incidence of CHD
7	Houtsmuller et al[8]	1981	Newly diagnosed T2DM	Yes	↓diabetic microvascular disease
8	The Finnish Mental Hospital Women Study[9]	1983	Hospital female patients	Maybe	↓ incidence of CHD (*P*=0.10)
9	DART[10]	1989	With MI	No	No effect on re-infarction and CHD death
10	The Minnesota Coronary Survey[11]	1989	From mental hospitals and nursing homes	No	No effect on MI and sudden death
11	STARS[12]	1992	With angina or past MI	Yes	↓severity of coronary stenoses
5	Reanalysis of Sydney Diet Heart Study[13]	2013	With CHD	No. Detrimental	↑ all-cause mortality (*P*=0.05), cardiovascular mortality (*P*=0.04), and CHD mortality (*P*=0.04)
10	Reanalysis of Minnesota Coronary Survey[14]	2016	From mental hospitals and nursing homes	No. Maybe detrimental	Partially recovered autopsy data suggested that the intervention may worsen the coronary atherosclerosis score and increase the death risk due to MI. In those aged ⩾65, intervention may increase the risk of death. A 22% higher risk of death was associated with each 30 mg/dL reduction in serum cholesterol.

↑, increase; ↓, decrease; CHD, coronary heart disease; DART: Diet and Reinfarction Trial; m, month; MRC, Medical Research Council; Pub, publication; PUFA, polyunsaturated fatty acid; STARS: St Thomas' Atheroma Regression Study; T2DM, type 2 diabetes; TC, total cholesterol; y, year.

References

1. Rose G, Thomson W, Williams R. Corn oil in treatment of ischaemic heart disease. *BMJ* 1965;**1**:1531.
2. Morris JN, al. e. Controlled trial of soya-bean oil in myocardial infarction. *Lancet* 1968;**2**:693-700.
3. Leren P. The effect of plasma-cholesterol-lowering diet in male survivors of myocardial infarction. A controlled clinical trial. *Bull N Y Acad Med* 1968;**44**:1012-1020.
4. DAYTON S, PEARCE ML, HASHIMOTO S, DIXON WJ, TOMIYASU U. A Controlled Clinical Trial of a Diet High in Unsaturated Fat in Preventing Complications of Atherosclerosis. *Circulation* 1969;**40**:II-1-II-63.
5. Pearce ML, Dayton S. Incidence of cancer in men on a diet high in polyunsaturated fat. *Lancet* 1971;**1**:464-467.
6. Woodhill JM, Palmer AJ, Leelarthaepin B, McGilchrist C, Blacket RB. Low fat, low cholesterol diet in secondary prevention of coronary heart disease. *Adv Exp Med Biol* 1978;**109**:317-330.
7. Turpeinen O, Karvonen MJ, Pekkarinen M, Miettinen M, Elosuo R, Paavilainen E. Dietary prevention of coronary heart disease: the Finnish Mental Hospital Study. *Int J Epidemiol* 1979;**8**:99-118.
8. Houtsmuller AJ, van Hal-Ferwerda J, Zahn KJ, Henkes HE. Favorable influences of linoleic acid on the progression of diabetic micro- and macroangiopathy in adult onset diabetes mellitus. *Prog Lipid Res* 1981;**20**:377-386.
9. Miettinen M, Turpeinen O, Karvonen M, Pekkarinen M, Paavilainen E, Elosuo R. Dietary prevention of coronary heart disease in women: the Finnish mental hospital study. *Int J Epidemiol* 1983;**12**:17-25.
10. Burr ML, Fehily AM, Gilbert JF, Rogers S, Holliday RM, Sweetnam PM, Elwood PC, Deadman NM. Effects of changes in fat, fish, and fibre intakes on death and myocardial reinfarction: diet and reinfarction trial (DART). *Lancet* 1989;**2**:757-761.
11. Frantz Jr I, Dawson EA, Ashman PL, Gatewood L, Bartsch G, Kuba K, Brewer E. Test of effect of lipid lowering by diet on cardiovascular risk. The Minnesota Coronary Survey. *Arteriosclerosis* 1989;**9**:129-135.
12. Watts G, Lewis B, Lewis E, Coltart D, Smith L, Swan A, Brunt J, Mann J. Effects on coronary artery disease of lipid-lowering diet, or diet plus cholestyramine, in the St Thomas' Atherosclerosis Regression Study (STARS). *Lancet* 1992;**339**:563-569.
13. Ramsden CE, Zamora D, Leelarthaepin B, Majchrzak-Hong SF, Faurot KR, Suchindran CM, Ringel A, Davis JM, Hibbeln JR. Use of dietary linoleic acid for secondary prevention of coronary heart disease and death: evaluation of recovered data from the Sydney Diet Heart Study and updated meta-analysis. *BMJ* 2013;**346**:e8707.
14. Ramsden CE, Zamora D, Majchrzak-Hong S, Faurot KR, Broste SK, Frantz RP, Davis JM, Ringel A, Suchindran CM, Hibbeln JR. Re-evaluation of the traditional diet-heart hypothesis: analysis of recovered data from Minnesota Coronary Experiment (1968-73). *BMJ* 2016;**353**:i1246

Supplementary Table 2. Intake levels of dietary polyunsaturated unsaturated fatty acids (PUFAs) and decrease in serum cholesterol in randomised controlled trials investigating the effect of dietary PUFAs on heart disease outcomes

Trial number	Trial name	% of calories from PUFAs		Decrease in serum cholesterol (relative to control)	
		Intervention	control	%	mg/dL
1	Rose Corn Oil Trial	29.5% [a]	NR	11.8%	30.5
2	MRC Soy Oil Trial	31.3% [b]	NR	16.6%	45.0
3	Oslo Diet-Heart Study	20.7%	NR	13.9%	41
4	Los Angeles Veterans	15.4%	4.0%	12.7%	28
5	Sydney Diet Heart Study	15.1%	8.9%	4.1%	11.4
6	The Finnish Mental Hospital Men Study	13.6%	4.6%	15%	41.4
7	Houtsmuller et al	18.4%	4.8%	NR	NR
8	The Finnish Mental Hospital Women Study	13.6%	4.6%	13%	35.2
10	DART	9%	7%	4.0%	10
	The Minnesota Coronary Survey	14.7%	NR	12.8 %	29
11	STARS	8%	NR	12.2%[c]	34.0[c]
	Mean	17.2%	5.7%	11.6%	30.6

NR: not reported. See Table S1 for other abbreviations and the reference for each trial.

[a] A daily intake of 80 g of corn oil was reported. The daily intake of total calories was assumed as 2,442 kcal, which was the mean of the following two trials published around a similar time (1965-1969): the Oslo Diet-Heart Study (2,387 kcal) and the Los Angeles Veterans study (2,496 kcal). The percentage of calories from PUFAs was calculated using the following formula: (grams of corn oil X 9 / total calorie intake in kcal) X 100.

[b] A daily intake of 85 g of soybean oil was reported. The daily intake of total calories was assumed as 2,442 kcal, as explained in the above footnote. The percentage of calories from PUFAs was calculated using the following formula: (grams of soybean oil X 9 / total calorie intake in kcal) X 100.

[c] The St Thomas' Atheroma Regression Study (STARS) measured cholesterol in the plasma instead of serum.

Supplementary Table 3. A 1-natural-log increase in percentage of calorie intake from n3 or n6 fatty acids and risk for heart disease mortality among 45,820 adults

Models	n3[a]		n6[b]	
	HR (95% CI)	*P* value	HR (95% CI)	*P* value
Overall (N=45,820)				
Model 1	0.77 (0.72-0.82)	<0.001	0.75 (0.69-0.81)	<0.001
Model 2	0.78 (0.73-0.83)	<0.001	0.74 (0.69-0.80)	<0.001
Model 3	0.95 (0.88-1.02)	0.152	0.90 (0.83-0.97)	0.008
Model 4	0.96 (0.89-1.03)	0.247	0.91 (0.84-0.99)	0.024
Participants with prior MI (N=1,541)				
Model 1	0.74 (0.65-0.85)	<0.001	0.81 (0.66-0.98)	0.032
Model 2	0.74 (0.64-0.85)	<0.001	0.79 (0.65-0.96)	0.016
Model 3	0.96 (0.82-1.13)	0.647	0.97 (0.80-1.19)	0.780
Model 4	0.95 (0.80-1.13)	0.555	0.95 (0.78-1.16)	0.612
Participants without prior MI (N=44,279)				
Model 1	0.77 (0.72-0.83)	<0.001	0.74 (0.67-0.80)	<0.001
Model 2	0.78 (0.73-0.84)	<0.001	0.73 (0.67-0.80)	<0.001
Model 3	0.94 (0.86-1.01)	0.104	0.87 (0.80-0.95)	0.003
Model 4	0.95 (0.87-1.03)	0.190	0.89 (0.82-0.98)	0.013

Abbreviations: CI, confidence interval; HR, hazard ratio; MI, myocardial infarction; MUFA, monounsaturated fatty acid; PUFA, polyunsaturated fatty acid; SFA, saturated fatty acid.

[a] n3 fatty acids included linolenic acid (18:3), stearidonic acid (18:4), eicosapentaenoic acid (20:5, EPA), docosapentaenoic acid (22:5, DPA), and docosahexaenoic acid (22:6, DHA).

[b] n6 fatty acids included linoleic acid (18:2) and arachidonic acid (20:4).

Model 1: adjusted for age; Model 2: adjusted for age, sex, and ethnicity; Model 3: adjusted for age, sex, ethnicity, obesity, poverty-income ratio, education, physical activity, alcohol consumption, smoking status, survey period, usual intake of protein (natural log transformed), and usual intake of carbohydrate (natural log transformed); Model 4: adjusted for all the factors in Model 3 plus systolic blood pressure (natural log transformed), total cholesterol (natural log transformed), and hemoglobin A_{1c} (natural log transformed).

Supplementary Table 4. A 1-natural-log higher usual intake of fatty acids in grams (*e.g.*, from 10 g to 27 g per day) and heart disease mortality risk among 45,820 adults

Models	SFA		MUFA		PUFA	
	HR (95% CI)	*P* value	HR (95% CI)	*P* value	HR (95% CI)	*P* value
Overall (N=45,820)						
Model 1	0.99 (0.90-1.09)	0.820	0.93 (0.84-1.02)	0.124	0.76 (0.69-0.83)	<0.001
Model 2	0.79 (0.72-0.88)	<0.001	0.73 (0.66-0.80)	<0.001	0.64 (0.59-0.71)	<0.001
Model 3	0.96 (0.85-1.10)	0.001	0.86 (0.76-0.99)	0.029	0.86 (0.77-0.96)	0.009
Model 4	1.00 (0.88-1.14)	0.957	0.90 (0.79-1.03)	0.117	0.89 (0.79-1.00)	0.041
Participants with prior MI (N=1,541)						
Model 1	0.85 (0.70-1.04)	0.119	0.82 (0.67-1.01)	0.067	0.73 (0.60-0.90)	0.003
Model 2	0.75 (0.61-0.93)	0.007	0.72 (0.58-0.89)	0.002	0.64 (0.52-0.79)	<0.001
Model 3	1.02 (0.75-1.38)	0.918	0.94 (0.69-1.28)	0.693	0.94 (0.72-1.23)	0.652
Model 4	1.06 (0.77-1.44)	0.734	0.94 (0.69-1.28)	0.681	0.93 (0.70-1.22)	0.577
Participants without prior MI (N=44,279)						
Model 1	1.03 (0.93-1.15)	0.578	0.95 (0.85-1.06)	0.353	0.77 (0.69-0.85)	<0.001
Model 2	0.86 (0.77-0.96)	0.006	0.77 (0.69-0.86)	<0.001	0.67 (0.60-0.74)	<0.001
Model 3	0.99 (0.86-1.15)	0.925	0.86 (0.75-1.00)	0.051	0.84 (0.74-0.95)	0.007
Model 4	1.02 (0.88-1.18)	0.775	0.90 (0.78-1.05)	0.183	0.87 (0.77-0.99)	0.035

Abbreviations: CI, confidence interval; HR, hazard ratio; MI, myocardial infarction; MUFA, monounsaturated fatty acid; PUFA, polyunsaturated fatty acid; SFA, saturated fatty acid.

Model 1: adjusted for age; Model 2: adjusted for age, sex, and ethnicity; Model 3: adjusted for age, sex, ethnicity, obesity, poverty-income ratio, education, physical activity, alcohol consumption, smoking status, survey period, usual intake of protein (natural log transformed), and usual intake of carbohydrate (natural log transformed); Model 4: adjusted for all the factors in Model 3 plus systolic blood pressure (natural log transformed), total cholesterol (natural log transformed), and hemoglobin A_{1c} (natural log transformed).

Supplementary Table 5. Sensitivity analysis of a 5% higher calorie intake from usual intake of fatty acids and heart disease mortality risk among 45,820 adults when comorbidities were treated as categorical variables instead of continuous variables

Models	SFA		MUFA		PUFA	
	HR (95% CI)	*P* value	HR (95% CI)	*P* value	HR (95% CI)	*P* value
Overall (N=45,820)						
Model 1	1.19 (1.11-1.28)	<0.001	1.13 (1.05-1.21)	0.002	0.81 (0.74-0.89)	<0.001
Model 2	1.17 (1.08-1.26)	<0.001	1.06 (0.99-1.14)	0.118	0.83 (0.75-0.92)	<0.001
Model 3	1.00 (0.93-1.08)	0.986	0.93 (0.86-1.01)	0.074	0.89 (0.81-0.97)	0.012
Model 4	1.02 (0.94-1.10)	0.617	0.94 (0.87-1.01)	0.103	0.90 (0.82-0.99)	0.031
Participants with prior MI (N=1,541)						
Model 1	1.12 (0.94-1.33)	0.198	1.10 (0.94-1.30)	0.245	0.94 (0.75-1.17)	0.571
Model 2	1.11 (0.93-1.33)	0.234	1.08 (0.91-1.27)	0.386	0.93 (0.75-1.17)	0.549
Model 3	1.01 (0.84-1.23)	0.892	0.95 (0.80-1.13)	0.590	0.95 (0.77-1.18)	0.649
Model 4	1.07 (0.89-1.30)	0.464	0.96 (0.81-1.13)	0.604	0.89 (0.72-1.11)	0.302
Participants without prior MI (N=44,279)						
Model 1	1.21 (1.12-1.31)	<0.001	1.12 (1.04-1.22)	0.005	0.79 (0.71-0.88)	<0.001
Model 2	1.20 (1.10-1.30)	<0.001	1.06 (0.97-1.15)	0.185	0.80 (0.72-0.90)	<0.001
Model 3	1.03 (0.94-1.12)	0.572	0.94 (0.86-1.02)	0.121	0.86 (0.78-0.96)	0.007
Model 4	1.03 (0.94-1.12)	0.526	0.94 (0.86-1.02)	0.132	0.89 (0.80-0.99)	0.035

Abbreviations: CI, confidence interval; HR, hazard ratio; MI, myocardial infarction; MUFA, monounsaturated fatty acid; PUFA, polyunsaturated fatty acid; SFA, saturated fatty acid.

Model 1: adjusted for age; Model 2: adjusted for age, sex, and ethnicity; Model 3: adjusted for age, sex, ethnicity, obesity, poverty-income ratio, education, physical activity, alcohol consumption, smoking status, survey period, usual intake of protein (natural log transformed), and usual intake of carbohydrate (natural log transformed); Model 4: adjusted for all the factors in Model 3 plus comorbidities including hypertension, hypercholesterolemia, and diabetes.

Supplementary Table 6. Sensitivity analysis of quartiles of percentage of calories from usual intake of fatty acids and heart disease mortality risk among 45,820 adults when comorbidities were treated as categorical instead of continuous variables

Quartile	SFA		MUFA		PUFA	
	HR[a] (95% CI)	*P* value	HR[a] (95% CI)	*P* value	HR[a] (95% CI)	*P* value
Overall (N=45,820)						
Q1	Reference		Reference		Reference	
Q2	1.01 (0.89-1.14)	0.910	0.90 (0.80-1.02)	0.105	0.96 (0.86-1.08)	0.476
Q3	1.02 (0.90-1.15)	0.790	1.04 (0.93-1.17)	0.497	0.90 (0.80-1.01)	0.067
Q4	1.08 (0.95-1.21)	0.242	0.92 (0.81-1.03)	0.157	0.90 (0.80-1.01)	0.063
Participants with prior MI (N=1,541)						
Q1	Reference		Reference		Reference	
Q2	1.02 (0.78-1.35)	0.873	1.03 (0.78-1.36)	0.822	1.03 (0.79-1.36)	0.817
Q3	1.20 (0.91-1.59)	0.191	1.01 (0.77-1.32)	0.957	0.83 (0.63-1.09)	0.174
Q4	1.15 (0.86-1.52)	0.349	1.00 (0.76-1.33)	0.998	0.91 (0.71-1.18)	0.483
Participants without prior MI (N=44,279)						
Q1	Reference		Reference		Reference	
Q2	1.01 (0.88-1.16)	0.893	0.87 (0.75-1.00)	0.044	0.94 (0.83-1.07)	0.331
Q3	1.00 (0.87-1.15)	0.985	1.04 (0.91-1.19)	0.562	0.90 (0.79-1.03)	0.128
Q4	1.09 (0.95-1.25)	0.203	0.92 (0.80-1.05)	0.219	0.87 (0.76-0.99)	0.028

Abbreviations: CI, confidence interval; HR, hazard ratio; MI, myocardial infarction; MUFA, monounsaturated fatty acid; PUFA, polyunsaturated fatty acid; Q, quartile; SFA, saturated fatty acid.

[a] Adjusted for age, sex, ethnicity, obesity, poverty-income ratio, education, physical activity, alcohol consumption, smoking status, survey period, usual intake of protein (natural log transformed), usual intake of carbohydrate (natural log transformed), and comorbidities including hypertension, hypercholesterolemia, and diabetes.

Supplementary Table 7. Sensitivity analysis of a 5% higher calorie intake from usual intake of fatty acids and heart disease mortality risk among 42,560 adults [a] when participants with missing data on systolic blood pressure, total cholesterol, or hemoglobin A_{1c} were excluded

Models	SFA		MUFA		PUFA	
	HR (95% CI)	*P* value	HR (95% CI)	*P* value	HR (95% CI)	*P* value
Overall (N=42,560)						
Model 1	1.19 (1.10-1.28)	<0.001	1.13 (1.05-1.22)	0.002	0.82 (0.74-0.91)	<0.001
Model 2	1.16 (1.08-1.26)	<0.001	1.06 (0.99-1.15)	0.114	0.84 (0.76-0.93)	0.001
Model 3	1.00 (0.92-1.08)	0.974	0.94 (0.87-1.02)	0.112	0.89 (0.80-0.98)	0.018
Model 4	1.01 (0.93-1.09)	0.824	0.96 (0.88-1.03)	0.254	0.91 (0.82-1.00)	0.058
Participants with prior MI (N=1,437)						
Model 1	1.11 (0.93-1.33)	0.252	1.08 (0.91-1.28)	0.355	0.96 (0.76-1.20)	0.699
Model 2	1.10 (0.92-1.32)	0.307	1.06 (0.89-1.25)	0.525	0.94 (0.75-1.19)	0.615
Model 3	0.98 (0.80-1.20)	0.845	0.96 (0.80-1.15)	0.622	0.91 (0.73-1.14)	0.403
Model 4	1.01 (0.83-1.24)	0.899	0.95 (0.80-1.14)	0.604	0.89 (0.71-1.11)	0.293
Participants without prior MI (N=41,123)						
Model 1	1.21 (1.12-1.32)	<0.001	1.13 (1.04-1.23)	0.004	0.80 (0.71-0.89)	<0.001
Model 2	1.20 (1.10-1.31)	<0.001	1.06 (0.98-1.16)	0.154	0.81 (0.72-0.91)	<0.001
Model 3	1.03 (0.94-1.12)	0.535	0.94 (0.86-1.03)	0.186	0.87 (0.78-0.97)	0.011
Model 4	1.03 (0.95-1.13)	0.485	0.96 (0.88-1.05)	0.381	0.89 (0.80-1.00)	0.044

Abbreviations: CI, confidence interval; HR, hazard ratio; MI, myocardial infarction; MUFA, monounsaturated fatty acid; PUFA, polyunsaturated fatty acid; SFA, saturated fatty acid.

[a] A total of 3,260 participants were excluded due to missing data on systolic blood pressure, total cholesterol, or hemoglobin A_{1c}. Therefore, the remaining 42,560 participants were included in the final analysis.

Model 1: adjusted for age; Model 2: adjusted for age, sex, and ethnicity; Model 3: adjusted for age, sex, ethnicity, obesity, poverty-income ratio, education, physical activity, alcohol consumption, smoking status, survey period, usual intake of protein (natural log transformed), and usual intake of carbohydrate (natural log transformed); Model 4: adjusted for all the factors in Model 3 plus systolic blood pressure (natural log transformed), total cholesterol (natural log transformed), and hemoglobin A_{1c} (natural log transformed).

Supplementary Table 8. Sensitivity analysis of quartiles of percentage of calories from usual intake of fatty acids and heart disease mortality risk among 42,560 adults [a] when participants with missing data on systolic blood pressure, total cholesterol, or hemoglobin A_{1c} were excluded

Quartile	SFA		MUFA		PUFA	
	HR [b] (95% CI)	*P* value	HR [b] (95% CI)	*P* value	HR [b] (95% CI)	*P* value
Overall (N=45,820)						
Q1	Reference		Reference		Reference	
Q2	1.01 (0.89-1.14)	0.891	1.07 (0.94-1.21)	0.315	0.99 (0.88-1.12)	0.896
Q3	1.01 (0.89-1.14)	0.913	1.00 (0.88-1.13)	0.977	0.89 (0.79-1.01)	0.062
Q4	1.05 (0.92-1.19)	0.473	1.12 (0.99-1.26)	0.070	0.91 (0.81-1.03)	0.122
Participants with prior MI (N=1,541)						
Q1	Reference		Reference		Reference	
Q2	0.95 (0.71-1.28)	0.749	1.01 (0.75-1.35)	0.947	1.06 (0.8-1.41)	0.671
Q3	0.92 (0.68-1.24)	0.575	1.10 (0.83-1.47)	0.505	0.91 (0.68-1.21)	0.505
Q4	1.08 (0.82-1.42)	0.592	0.97 (0.73-1.28)	0.801	0.91 (0.69-1.19)	0.476
Participants without prior MI (N=44,279)						
Q1	Reference		Reference		Reference	
Q2	0.92 (0.80-1.06)	0.257	1.05 (0.91-1.20)	0.537	0.96 (0.84-1.1)	0.580
Q3	0.93 (0.82-1.07)	0.329	0.97 (0.84-1.12)	0.674	0.89 (0.77-1.02)	0.088
Q4	0.91 (0.80-1.04)	0.170	1.10 (0.97-1.26)	0.139	0.87 (0.76-0.99)	0.040

Abbreviations: CI, confidence interval; HR, hazard ratio; MI, myocardial infarction; MUFA, monounsaturated fatty acid; PUFA, polyunsaturated fatty acid; Q, quartile; SFA, saturated fatty acid.

[a] A total of 3,260 participants were excluded due to missing data on systolic blood pressure, total cholesterol, or hemoglobin A_{1c}. Therefore, the remaining 42,560 participants were included in the final analysis.

[b] Adjusted for age, sex, ethnicity, obesity, poverty-income ratio, education, physical activity, alcohol consumption, smoking status, survey period, usual intake of protein (natural log transformed), usual intake of carbohydrate (natural log transformed), systolic blood pressure (natural log transformed), total cholesterol (natural log transformed), and hemoglobin A_{1c} (natural log transformed).

Supplementary Table 9. Sensitivity analysis of a 5% higher calorie intake from fatty acids and heart disease mortality risk among 45,820 adults when usual intake of fatty acids was replaced with mean intake of fatty acids

Models	SFA		MUFA		PUFA	
	HR (95% CI)	*P* value	HR (95% CI)	*P* value	HR (95% CI)	*P* value
Overall (N=45,820)						
Model 1	1.11 (1.05-1.17)	<0.001	1.07 (1.01-1.13)	0.016	0.84 (0.79-0.90)	<0.001
Model 2	1.08 (1.02-1.14)	0.006	1.01 (0.96-1.07)	0.745	0.84 (0.78-0.90)	<0.001
Model 3	1.00 (0.95-1.06)	0.998	0.95 (0.90-1.01)	0.093	0.92 (0.86-0.98)	0.014
Model 4	1.01 (0.95-1.07)	0.773	0.97 (0.91-1.02)	0.227	0.94 (0.87-1.00)	0.049
Participants with prior MI (N=1,541)						
Model 1	1.05 (0.93-1.20)	0.416	1.04 (0.93-1.18)	0.482	0.92 (0.79-1.08)	0.306
Model 2	1.04 (0.92-1.18)	0.547	1.02 (0.90-1.15)	0.778	0.91 (0.77-1.06)	0.220
Model 3	1.01 (0.88-1.15)	0.924	0.97 (0.86-1.09)	0.571	0.97 (0.83-1.12)	0.646
Model 4	1.03 (0.90-1.17)	0.723	0.96 (0.85-1.08)	0.500	0.96 (0.82-1.11)	0.560
Participants without prior MI (N=44,279)						
Model 1	1.13 (1.06-1.20)	<0.001	1.07 (1.01-1.14)	0.028	0.83 (0.77-0.89)	<0.001
Model 2	1.11 (1.04-1.18)	0.001	1.01 (0.95-1.08)	0.705	0.82 (0.76-0.89)	<0.001
Model 3	1.02 (0.96-1.09)	0.503	0.96 (0.90-1.02)	0.191	0.90 (0.84-0.97)	0.008
Model 4	1.03 (0.97-1.09)	0.412	0.98 (0.92-1.04)	0.420	0.92 (0.86-0.99)	0.034

Abbreviations: CI, confidence interval; HR, hazard ratio; MI, myocardial infarction; MUFA, monounsaturated fatty acid; PUFA, polyunsaturated fatty acid; SFA, saturated fatty acid.

Model 1: adjusted for age; Model 2: adjusted for age, sex, and ethnicity; Model 3: adjusted for age, sex, ethnicity, obesity, poverty-income ratio, education, physical activity, alcohol consumption, smoking status, survey period, mean protein intake, and mean carbohydrate intake; Model 4: adjusted for all the factors in Model 3 plus systolic blood pressure (natural log transformed), total cholesterol (natural log transformed), and hemoglobin A_{1c} (natural log transformed).

Supplementary Table 10. Sensitivity analysis of a 5% higher calorie intake from usual intake of fatty acids and heart disease mortality risk among 34,501 adults [a] with further adjustment for C-reactive protein

Models	SFA		MUFA		PUFA	
	HR (95% CI)	*P* value	HR (95% CI)	*P* value	HR (95% CI)	*P* value
Overall (N=34,501)						
Model 1	1.02 (0.94-1.11)	0.624	0.94 (0.87-1.02)	0.145	0.89 (0.81-0.99)	0.024
Model 2	1.01 (0.93-1.10)	0.798	0.94 (0.87-1.02)	0.156	0.90 (0.81-0.99)	0.034
Participants with prior MI (N=1,143)						
Model 1	1.06 (0.86-1.31)	0.575	0.95 (0.79-1.13)	0.540	0.85 (0.68-1.08)	0.182
Model 2	1.04 (0.85-1.28)	0.703	0.96 (0.8-1.14)	0.616	0.83 (0.66-1.05)	0.113
Participants without prior MI (N=33,358)						
Model 1	1.03 (0.94-1.13)	0.495	0.95 (0.87-1.03)	0.224	0.88 (0.79-0.99)	0.031
Model 2	1.02 (0.94-1.12)	0.607	0.95 (0.87-1.04)	0.225	0.89 (0.80-1.00)	0.045

Abbreviations: CI, confidence interval; HR, hazard ratio; MI, myocardial infarction; MUFA, monounsaturated fatty acid; PUFA, polyunsaturated fatty acid; SFA, saturated fatty acid.

[a] 11,319 out of the 45,820 participants did not have C-reactive protein values and were excluded from the analysis. Therefore, 34,501 participants were included in the final analysis.

Model 1: adjusted for age, sex, ethnicity, obesity, poverty-income ratio, education, physical activity, alcohol consumption, smoking status, survey period, usual intake of protein (natural log transformed), usual intake of carbohydrate (natural log transformed), hypertension, hypercholesterolemia, and diabetes.

Model 2: adjusted for all the factors in Model 1 plus C-reactive protein.

Supplementary Table 11. Sensitivity analysis of a 5% higher calorie intake from usual intake of fatty acids and risk for heart disease mortality among 45,414 [a] adults when total energy intake was adjusted and BMI was treated as a continuous variable.

Models	SFA		MUFA		PUFA	
	HR (95% CI)	*P* value	HR (95% CI)	*P* value	HR (95% CI)	*P* value
Overall (N=45,414)						
Model 1	1.18 (1.09-1.27)	<0.001	1.12 (1.04-1.20)	0.004	0.82 (0.74-0.90)	<0.001
Model 2	1.16 (1.07-1.25)	<0.001	1.05 (0.98-1.13)	0.180	0.84 (0.76-0.93)	0.001
Model 3	1.01 (0.92-1.10)	0.859	0.91 (0.83-1.01)	0.066	0.91 (0.82-1.00)	0.057
Model 4	1.03 (0.94-1.12)	0.522	0.94 (0.85-1.03)	0.197	0.92 (0.83-1.02)	0.106
Participants with prior MI (N=1,510)						
Model 1	1.13 (0.94-1.34)	0.188	1.11 (0.94-1.31)	0.208	0.96 (0.77-1.21)	0.75
Model 2	1.12 (0.94-1.34)	0.220	1.09 (0.92-1.29)	0.316	0.96 (0.77-1.20)	0.741
Model 3	0.99 (0.79-1.26)	0.955	0.92 (0.72-1.16)	0.465	0.97 (0.77-1.24)	0.825
Model 4	1.03 (0.81-1.31)	0.788	0.90 (0.70-1.14)	0.367	0.95 (0.75-1.21)	0.676
Participants without prior MI (N=43,904)						
Model 1	1.20 (1.10-1.30)	<0.001	1.11 (1.02-1.20)	0.016	0.79 (0.71-0.88)	<0.001
Model 2	1.18 (1.08-1.28)	<0.001	1.04 (0.96-1.14)	0.307	0.81 (0.72-0.90)	<0.001
Model 3	1.03 (0.94-1.14)	0.523	0.91 (0.81-1.01)	0.079	0.87 (0.78-0.98)	0.019
Model 4	1.05 (0.95-1.16)	0.323	0.94 (0.84-1.05)	0.255	0.89 (0.79-1.00)	0.042

Abbreviations: BMI, body mass index; CI, confidence interval; HR, hazard ratio; MI, myocardial infarction; MUFA, monounsaturated fatty acid; PUFA, polyunsaturated fatty acid; SFA, saturated fatty acid.

[a] A total of 406 participants who did not have BMI data were excluded. Therefore 45,414 participants were included in the analysis.

Model 1: adjusted for age; Model 2: adjusted for age, sex, and ethnicity; Model 3: adjusted for age, sex, ethnicity, BMI (natural log transformed), poverty-income ratio, education, physical activity, alcohol consumption, smoking status, survey period, usual intake of protein (natural log transformed), usual intake of carbohydrate (natural log transformed), and mean total energy intake (natural log transformed); Model 4: adjusted for all the factors in Model 3 plus systolic blood pressure (natural log transformed), total cholesterol (natural log transformed), and hemoglobin A_{1c} (natural log transformed).

Supplementary Table 12. Sensitivity analysis of a 5% higher calorie intake from usual intake of fatty acids and risk for heart disease mortality among 45,820 adults with further adjustment for the use of aspirin and statin

Models	SFA		MUFA		PUFA	
	HR (95% CI)	*P* value	HR (95% CI)	*P* value	HR (95% CI)	*P* value
Overall (N=45,820)						
Model 1	1.02 (0.94-1.10)	0.673	0.95 (0.88-1.03)	0.224	0.91 (0.83-1.00)	0.048
Model 2	1.02 (0.94-1.1)	0.671	0.95 (0.88-1.03)	0.225	0.91 (0.83-1.00)	0.047
Model 3	1.02 (0.95-1.1)	0.588	0.96 (0.89-1.03)	0.241	0.91 (0.83-1.00)	0.044
Model 4	1.02 (0.95-1.1)	0.588	0.96 (0.89-1.03)	0.242	0.91 (0.82-1.00)	0.044
Participants with prior MI (N=1,541)						
Model 1	1.05 (0.86-1.27)	0.652	0.95 (0.80-1.13)	0.540	0.94 (0.75-1.16)	0.546
Model 2	1.04 (0.86-1.27)	0.658	0.95 (0.80-1.13)	0.532	0.93 (0.75-1.16)	0.537
Model 3	1.04 (0.86-1.26)	0.673	0.95 (0.80-1.13)	0.534	0.93 (0.75-1.16)	0.541
Model 4	1.04 (0.86-1.26)	0.676	0.95 (0.79-1.13)	0.526	0.93 (0.75-1.16)	0.533
Participants without prior MI (N=44,279)						
Model 1	1.04 (0.95-1.13)	0.413	0.96 (0.88-1.05)	0.340	0.89 (0.80-0.99)	0.032
Model 2	1.04 (0.95-1.13)	0.413	0.96 (0.88-1.05)	0.340	0.89 (0.80-0.99)	0.031
Model 3	1.04 (0.95-1.13)	0.396	0.96 (0.88-1.05)	0.346	0.89 (0.80-0.99)	0.031
Model 4	1.04 (0.95-1.13)	0.396	0.96 (0.88-1.05)	0.346	0.89 (0.80-0.99)	0.030

Abbreviations: CI, confidence interval; HR, hazard ratio; MI, myocardial infarction; MUFA, monounsaturated fatty acid; PUFA, polyunsaturated fatty acid; SFA, saturated fatty acid.

Model 1: adjusted for age, sex, ethnicity, obesity, poverty-income ratio, education, physical activity, alcohol consumption, smoking status, survey period, usual intake of protein (natural log transformed), usual intake of carbohydrate (natural log transformed), systolic blood pressure (natural log transformed), total cholesterol (natural log transformed), and hemoglobin A_{1c} (natural log transformed); Model 2: adjusted for all the factors in Model 1 plus use of aspirin (yes or no); Model 3: adjusted for all the factors in Model 1 plus use of statin (yes or no); Model 4: adjusted for all the factors in Model 1 plus use of aspirin (yes or no) and use of statin (yes or no).

Supplementary Table 13. Sensitivity analysis of a 5% higher calorie intake from usual intake of fatty acids and risk for heart disease mortality among 42,084[a] adults when those who had a mean total caloric intake of <500 or >3,500 kcal/day were excluded

Models	SFA		MUFA		PUFA	
	HR (95% CI)	*P* value	HR (95% CI)	*P* value	HR (95% CI)	*P* value
Overall (N=42,084)						
Model 1	1.20 (1.12-1.29)	<0.001	1.14 (1.06-1.23)	0.001	0.79 (0.72-0.88)	<0.001
Model 2	1.18 (1.09-1.27)	<0.001	1.07 (0.99-1.16)	0.079	0.82 (0.74-0.90)	<0.001
Model 3	1.01 (0.93-1.09)	0.842	0.94 (0.87-1.02)	0.140	0.88 (0.80-0.97)	0.010
Model 4	1.02 (0.95-1.11)	0.587	0.96 (0.89-1.04)	0.305	0.90 (0.81-0.99)	0.032
Participants with prior MI (N=1,463)						
Model 1	1.13 (0.94-1.34)	0.189	1.07 (0.91-1.27)	0.408	0.92 (0.74-1.15)	0.473
Model 2	1.12 (0.94-1.34)	0.213	1.05 (0.89-1.24)	0.580	0.92 (0.73-1.15)	0.460
Model 3	1.02 (0.84-1.24)	0.844	0.93 (0.78-1.12)	0.452	0.93 (0.75-1.16)	0.517
Model 4	1.05 (0.86-1.28)	0.642	0.92 (0.77-1.10)	0.378	0.92 (0.73-1.15)	0.439
Participants without prior MI (N=40,621)						
Model 1	1.23 (1.13-1.33)	<0.001	1.14 (1.05-1.24)	0.002	0.77 (0.69-0.87)	<0.001
Model 2	1.21 (1.11-1.31)	<0.001	1.07 (0.99-1.17)	0.103	0.78 (0.70-0.88)	<0.001
Model 3	1.03 (0.95-1.13)	0.471	0.95 (0.87-1.04)	0.262	0.86 (0.77-0.95)	0.005
Model 4	1.04 (0.95-1.14)	0.363	0.97 (0.89-1.06)	0.518	0.88 (0.79-0.98)	0.020

Abbreviations: CI, confidence interval; HR, hazard ratio; MI, myocardial infarction; MUFA, monounsaturated fatty acid; PUFA, polyunsaturated fatty acid; SFA, saturated fatty acid.

[a] A total of 3,736 participants who had a mean total caloric intake of <500 (N=278) or >3,500 kcal/day (N=3,458) were excluded. Therefore 42,084 participants were included in the analysis.

Model 1: adjusted for age; Model 2: adjusted for age, sex, and ethnicity; Model 3: adjusted for age, sex, ethnicity, obesity, poverty-income ratio, education, physical activity, alcohol consumption, smoking status, survey period, usual intake of protein (natural log transformed), and usual intake of carbohydrate (natural log transformed); Model 4: adjusted for all the factors in Model 3 plus systolic blood pressure (natural log transformed), total cholesterol (natural log transformed), and hemoglobin A_{1c} (natural log transformed).

Supplementary Table 14. Sensitivity analysis of a 5% higher calorie intake from usual intake of fatty acids and risk for heart disease mortality among 45,820 adults when total cholesterol was replaced by LDL cholesterol for adjustment

Models	SFA		MUFA		PUFA	
	HR (95% CI)	*P* value	HR (95% CI)	*P* value	HR (95% CI)	*P* value
Overall (N=45,820)						
Model 1	1.02 (0.94-1.10)	0.673	0.95 (0.88-1.03)	0.224	0.91 (0.83-1.00)	0.048
Model 2	1.02 (0.94-1.10)	0.687	0.95 (0.88-1.03)	0.209	0.91 (0.83-1.00)	0.048
Participants with prior MI (N=1,541)						
Model 1	1.05 (0.86-1.27)	0.652	0.95 (0.80-1.13)	0.540	0.94 (0.75-1.16)	0.546
Model 2	1.05 (0.86-1.27)	0.655	0.95 (0.80-1.13)	0.534	0.94 (0.75-1.17)	0.559
Participants without prior MI (N=44,279)						
Model 1	1.04 (0.95-1.13)	0.413	0.96 (0.88-1.05)	0.340	0.89 (0.80-0.99)	0.032
Model 2	1.04 (0.95-1.13)	0.421	0.96 (0.88-1.04)	0.321	0.89 (0.80-0.99)	0.031

Abbreviations: CI, confidence interval; HR, hazard ratio; LDL, low-density lipoprotein; MI, myocardial infarction; MUFA, monounsaturated fatty acid; PUFA, polyunsaturated fatty acid; SFA, saturated fatty acid.

Model 1: adjusted for age, sex, ethnicity, obesity, poverty-income ratio, education, physical activity, alcohol consumption, smoking status, survey period, usual intake of protein (natural log transformed), usual intake of carbohydrate (natural log transformed), systolic blood pressure (natural log transformed), total cholesterol (natural log transformed), and hemoglobin A_{1c} (natural log transformed); Model 2: adjusted for all the factors in Model 1 except that total cholesterol (natural log transformed) was replaced by LDL cholesterol.

Supplementary Table 15. Major food sources of fatty acids

SFA	MUFA	PUFA
Cheese	Egg	Mayonnaise
Egg	Beef	Egg
Beef	Sausage	Fish
Catfish	Chicken	French fries
Lasagna	Pork	Roll
Roll	Cheese	Sunflower seed
French fries	Pizza	Muffin
Cookie	Cake	Granola
Fish	Peanut butter	Chicken
Ice cream	Almond	Cake
Mayonnaise	French fries	Flax seed
Hot dog	Milk	Hot dog
Cake	Ice cream	Beef
Cream	Cookie	Cheese
Chicken	Fish	Nuts

MUFA, monounsaturated fatty acid; PUFA, polyunsaturated fatty acid; SFA, saturated fatty acid.
